# Individual differences in the attentional modulation of the human auditory brainstem response to speech inform on speech-in-noise deficits

**DOI:** 10.1038/s41598-019-50773-1

**Published:** 2019-10-01

**Authors:** Marina Saiz-Alía, Antonio Elia Forte, Tobias Reichenbach

**Affiliations:** 1 Department of Bioengineering and Centre for Neurotechnology, Imperial College London, South Kensington Campus, SW7 2AZ London, UK; 2 John A. Paulson School of Engineering and Applied Sciences, Harvard University, 29 Oxford St, Cambridge, MA 02138 USA

**Keywords:** Midbrain, Scientific data

## Abstract

People with normal hearing thresholds can nonetheless have difficulty with understanding speech in noisy backgrounds. The origins of such supra-threshold hearing deficits remain largely unclear. Previously we showed that the auditory brainstem response to running speech is modulated by selective attention, evidencing a subcortical mechanism that contributes to speech-in-noise comprehension. We observed, however, significant variation in the magnitude of the brainstem’s attentional modulation between the different volunteers. Here we show that this variability relates to the ability of the subjects to understand speech in background noise. In particular, we assessed 43 young human volunteers with normal hearing thresholds for their speech-in-noise comprehension. We also recorded their auditory brainstem responses to running speech when selectively attending to one of two competing voices. To control for potential peripheral hearing deficits, and in particular for cochlear synaptopathy, we further assessed noise exposure, the temporal sensitivity threshold, the middle-ear muscle reflex, and the auditory-brainstem response to clicks in various levels of background noise. These tests did not show evidence for cochlear synaptopathy amongst the volunteers. Furthermore, we found that only the attentional modulation of the brainstem response to speech was significantly related to speech-in-noise comprehension. Our results therefore evidence an impact of top-down modulation of brainstem activity on the variability in speech-in-noise comprehension amongst the subjects.

## Introduction

Understanding speech in noisy backgrounds such as other competing speakers is a challenging task at which humans excel^1,2^ It requires the separation of different sound sources, selective attention to the target speaker, and the processing of degraded signals^3–5^ Hearing impairment such as resulting from noise exposure often leads to an increase of hearing thresholds, a reduction in the information conveyed about a sound to the central auditory system, and thus to greater difficulty in understanding speech in noise^6–8^ However, even listeners with normal hearing thresholds can have problems with understanding speech in noisy environments^9,10^.

An extensive neural network of efferent fibers can feed information from the central auditory cortex back to the auditory brainstem and even to the cochlea^11,12^. Research on the role of these neural feedback loops for speech-in-noise listening has mostly focused on the medial olivocochlear reflex (MOCR), in which stimulation of the medial olivocochlear fibers that synapse on the outer hair cells in the cochlea reduces cochlear amplification across a wide frequency band^13^. Computational modelling as well as animal studies have shown that such reduced broad-band amplification can improve the signal-to-noise ratio of a transient signal embedded in background noise^14–17^. However, it remains debated whether the reduction of cochlear amplification through the MOCR contributes to better speech-in-noise comprehension in humans: some studies found evidence for this hypothesis^18–22^ whereas others did not^23,24^ and yet others found the opposite behaviour^25,26^.

We recently demonstrated a neural mechanism for listening in noisy backgrounds that involves the extensive efferent connections between the central auditory cortex and the brainstem^27–29^. In particular, we devised a mathematical method for measuring the human brainstem response to the periodicity in the voiced parts of speech, the temporal fine structure, and showed that this subcortical response was stronger when a speaker was attended than when he or she was ignored^30,31^. Importantly, and different from the MOCR, this attentional modulation did not occur across a broad frequency band but instead relied on the frequency-specificity of the brainstem response to the temporal fine structure. As another difference to the MOCR, the response was measured from the brainstem instead of from the cochlea.

Because we observed significant individual differences in the strength of the attentional modulation of the brainstem response to speech, we wondered if this was related to the individual’s ability to understand speech in noise. In particular, we hypothesized that a larger attentional modulation might lead to better speech-in-noise comprehension. Alternatively, we hypothesized that a larger attentional modulation might indicate larger difficulty with speech-in-noise comprehension and therefore worse performance in speech-in-noise listening.

A potential source of variation in the brainstem response to speech, as well as in speech-in-noise comprehension, is cochlear synaptopathy, a loss of synaptic connection between the auditory-nerve fibers and the mechanosensitive hair cells in the inner ear^32^. Because synapses of supra-threshold auditory-nerve fibers are predominantly affected, the condition does not lead to elevated hearing thresholds and has therefore also been referred to as hidden hearing loss^33,34^. To investigate our hypotheses regarding a correlation between the attentional modulation of the brainstem response to speech and speech-in-noise comprehension, we therefore sought to control for cochlear synaptopathy.

The most direct non-invasive measure of auditory-nerve activity in humans, and therefore potentially of cochlear synaptopathy, is wave I of the click-evoked auditory brainstem response. Animal studies have indeed shown that cochlear synaptopathy leads to a reduction in the amplitude of wave I. A reduced wave I has also been found to correlate with noise exposure of human subjects in some studies^35^ although not in others^36,37^. A difficulty with wave I as a clinical measure is, however, its significant variability across individual subjects, suggesting a need for more robust measure of cochlear synaptopathy. Rodent studies in conjunction with investigations on human subjects have suggested that the latency shift of the larger wave V, obtained from presenting clicks in varying levels of background noise, informs on cochlear synaptopathy^38,39^. Because cochlear synaptopathy probably decreases the timing precision of spikes in the auditory nerve, people with the condition should perform worse on low-level auditory tasks that rely on timing information, such as detecting small interaural timing differences^40^. Moreover, the middle-ear muscle reflex has been shown to be reduced by cochlear synaptopathy in rodents and may therefore serve as a clinical measure of this peripheral hearing impairment as well^41,42^. Here we have included the threshold for interaural timing differences, the latency shift of wave V, and the threshold of the middle-ear muscle reflex to control for cochlear synaptopathy.

## Materials and Methods

### Participants

43 healthy young volunteers with an age of 24 ± 3 years (mean and standard deviation), seventeen of which were female, were recruited from Imperial College London. All subjects were native English speakers, had no history of hearing or neurological impairments, and provided written informed consent. The experimental procedures were approved by the Imperial College Research Ethics Committee, and were performed in accordance with all relevant guidelines and regulations. Informed consent was obtained from all participants.

### Test environment

All testing was carried out in a sound-proof, electrically-insulated and semi-anechoic room. A personal computer outside the room controlled the audio presentation and data acquisition. Sound stimuli were presented at a sampling frequency of 44.1 kHz through a high-performance sound card (Xonar Essence STX, Asus, U.S.A.). They were delivered through insert earphones (ER-3C, Etymotic, U.S.A.). Sound intensity was calibrated with an ear simulator (Type 4157, Brüel & Kjaer, Denmark). Unless otherwise stated, the sound was presented diotically.

### Pure-tone audiometry

We measured audiometric thresholds at 250 Hz, 500 Hz, 1 kHz, 1.5 kHz, 2 kHz, 3 kHz, 4 kHz, 6 kHz and 8 kHz. The thresholds were assessed behaviourally using Otosure (Amplivox, U.K.).

### Noise exposure

Lifetime noise exposure was estimated using a structured interview^43^. This interview identified different sources of recreational or occupational exposure to noise of a high level, exceeding 80 dBA. The corresponding sound level was estimated through a list of the most common activities involving high noise exposure, such as visiting clubs with amplified music, attending events with live amplified music, listening to music through earphones, or riding the tube. For each free-field activity, the sound level was computed based on the vocal effort required to hold a conversation at a distance of 1.2 m. Reported vocal effort was converted to dBA using a speech communication table^43^. The resulting noise exposure was then quantified by the units of noise exposure *U*:

$$U=\frac{T}{2080}\ast {10}^{\frac{L-A-90}{10}}$$

in which *T* is the total exposure time in hours, *L* is the noise level in dBA, and *A* is the attenuation of ear protection in dBA^44^. The measure *U* is proportional to the total energy of exposure (above 80 dBA). The lifetime noise exposure of a subject followed from adding the noise exposures that resulted from each source the volunteer had been exposed to.

### Speech-in-noise perception

The ability of each participant to understand speech in noise was quantified using the 50% speech reception threshold for sentences in noise (SRTn), that is, the signal-to-noise ratio (SNR) at which the participant could correctly identify 50% of words in a given sentence that was embedded in background noise. We employed semantically unpredictable sentences spoken by a female speaker together with four-talker babble noise. The target speech was presented at 70 dBA while the SNR varied over trials.

We implemented an adaptive procedure, a fixed step staircase^45^. After listening to a sentence the subject repeated what they understood. The verbal response was recorded with a microphone and evaluated through automatic speech recognition (Google Speech API). The noise level of the following sentence was adapted based on the subject’s response.

All participants first listened to ten sentences with different SNRs, ranging from 12 to −5 dB, in order to familarise themselves with the task. The adaptive procedure then started with an initial SNR of 10 dB. The SNR was changed by a step size of 3 dB during the first four reversals, and the step size was decreased to 1 dB for the next twelve reversals. The SRTn was computed as the mean of the last six reversals. It took about 15 minutes to estimate the SRTn for a subject.

### Sensitivity to interaural timing differences (ITD)

To obtain a binaural measure of temporal coding, we assessed the threshold for detecting small timing differences in sound presented to the left and to the right ear. In particular, we employed a 4 kHz tone that was amplitude-modulated at 50 Hz^38,46^. The carrier phase was identical in the two ears, but an ITD was applied to the envelope so that the sound to the right ear was leading. The signals were embedded in noise that extended from 20 Hz to 20 kHz and that had a notch around the carrier frequency of 4 kHz, with a bandwidth set to the equivalent rectangular bandwidth (ERB) of a 4 kHz channel (i.e. 456.46 Hz). We employed different noise for each trial. The noise level was chosen such that the resulting signal had an SNR of 10 dB (broadband rms). The sounds were presented at 80 dB SPL.

The threshold for detecting the ITDs was determined using a three-cue, two-alternative forced-choice adaptive procedure. The first of the three consecutive sounds always contained a stimulus with an ITD of 0 μs and served as reference. Either the second or the third sound, at equal probability, contained an ITD that was not zero. The listener’s task was to detect and identify the sound with the non-vanishing ITD. The initial ITD of 1,200 μs was iteratively updated using a two-up one-down procedure^47^. The initial step size of 100 μs was halved after five reversals. A total of ten reversals were used and the threshold was calculated from the last five reversals. Up to 30 training trials with the initial ITD were performed before the start of the adaptive procedure to familiarize the participant with the task. The exercise was, however, nonetheless challenging to some subjects, and eleven volunteers were unable to perform it. The results from this measure should thus be interpreted with caution as they may not generalize to the population. It took at most 15 minutes to carry out the test for an individual subject.

### Click-evoked auditory-brainstem responses in noise

We measured auditory brainstem responses from five passive Ag/AgCl electrodes (Multitrode, BrainProducts, Germany). Two electrodes were positioned at the cranial vertex (Cz), two further electrodes on the left and right mastoid processes, and the remaining electrode served as ground and was positioned on the forehead. We lowered the impedance between each electrode and the skin to below 5 kΩ using abrasive electrolyte-gel (Abralyt HiCl, Easycap, Germany). The electrode on the left mastoid, at the cranial vertex and the ground electrode were connected to a bipolar amplifier with low-level noise and a gain of 50 (EP-PreAmp, BrainProducts, Germany). The remaining two electrodes were connected to a second identical bipolar amplifier. The output from both bipolar amplifiers was fed into an integrated amplifier (actiCHamp, BrainProducts, Germany). The recordings where thereby low-pass filtered through a hardware anti-aliasing filter with a corner frequency of 4.9 kHz, and were sampled at 25 kHz. The audio signals were measured by the integrated amplifier as well, using an acoustic adapter (Acoustical Stimulator Adapter and StimTrak, BrainProducts, Germany). All data were acquired through PyCorder (BrainProducts, Germany). The simultaneous measurement of the audio signal and the brainstem response from the integrated amplifier was employed to temporally align both signals to a precision of less than 40 µs, the inverse of the sampling rate (25 kHz). A delay of 1 ms of the acoustic signal produced by the earphones was taken into account.

We presented the volunteers with clicks of 80 dB peSPL (IEC 60645-3 peSPL calibration method) that were embedded in four different levels of broadband noise: 42 dB SPL, 52 dB SPL, 62 dB SPL and 72 dB SPL^38^. For each noise level we presented 3,000 clicks in blocks of 500 clicks, with equal numbers of rarefaction and condensation clicks per condition. The order in which the blocks with different polarities and the different noise levels were presented was randomized across participants. Clicks were presented at a rate of 10 Hz with 20 ms interclick jitter to avoid any stationary interference, such as from electrical noise. The recorded data were band-pass filtered between 100 and 2,000 Hz (4th order Butterworth IIR, 25 Hz and 500 Hz transition bands for low and high cutoff frequencies, respectively). The data was divided into segments that ranged from −5 to 10 ms relative to the onset of each click. We then computed the average response across the two channels from both hemispheres and averaged over the different segments to obtain the ABR waveform. All participants except one showed remarkably clear ABR waveforms, with the exception of the noise level of 72 dB SPL where wave V was occasionally not identifiable. These recordings took around 30 minutes per subject, including the electrode setup.

The latency of wave V was then obtained for each noise level as the peak amplitude between the delays of 6–10 ms. The peak was considered as significant if it exceeded the 95% percentile of the recording’s noise floor, which was established from the delays of −5 to 0 ms, for each noise level. This criterion excluded wave V in the 72 dB SPL condition for seven subjects. The latency change with increasing noise was then computed by a linear fit of the wave V latency versus the noise level.

### Middle-ear muscle reflex (MEMR)

Middle-ear muscle reflex thresholds were measured with a GSI Tympstar diagnostic middle-ear analyzer using 226 Hz probe tone. Stimuli were ipsilateral pulsed pure tones of frequencies of 1 kHz and 4 kHz, presented in the left ear (1.5 s on and off time). Reflex thresholds were determined using changes in middle-ear compliance following the presentation of the elicitor. A reflex response was defined as a reduction in compliance of 0.02 mmho or greater over two consecutive trials, to ensure that the response was not artefactual. For each tone, the stimulus level started at 75 dB and increased in 2 dB steps until the threshold criterion was reached. The MEMR threshold was then computed as the difference between threshold responses at 4 kHz and at 1 kHz. This differential measure has been suggested to provide increased sensitivity to noise-induced damage, which predominantly affects the 3–6 kHz region^44^.

Three participants showed unstable compliance responses with atypical morphology due to a poor fit of the probe. We therefore tested their right ear instead.

### Auditory brainstem responses to speech (speech-ABR) and attentional modulation

Auditory brainstem responses to speech were measured through the setup described in the subsection on click-evoked brainstem recordings. Subjects listened to two competing voices.

Samples of continuous speech from a male and a female speaker were obtained from publicly available audiobooks (https://librivox.org). In particular, we used extracts from “*Tales of Troy: Ulysses the sacker of cities*” and “*The green forest fairy book*”, narrated by James K. White, as well as chapters from “*The children of Odin*”, read by Elizabeth Klett. All samples had a duration of at least two minutes and ten seconds. To construct speech samples with two competing speakers, samples from the male and from the female speaker were normalized to the same root-mean-square amplitude and then superimposed. Stimuli were delivered at 72 dBA.

The experimental design employed two conditions, attention to the female voice and attention to the male voice. For attending the female voice, subjects were asked to listen to the female speaker and to ignore the male one, and *vice versa* for the other condition. For each condition we employed four samples, yielding around ten minutes of recording per condition. The order of the presentation of the different conditions was randomized across participants. Comprehension questions were asked at the end of each part in order to verify the subject’s attention to the corresponding story. All subjects answered the questions correctly.

**Figure 1:**
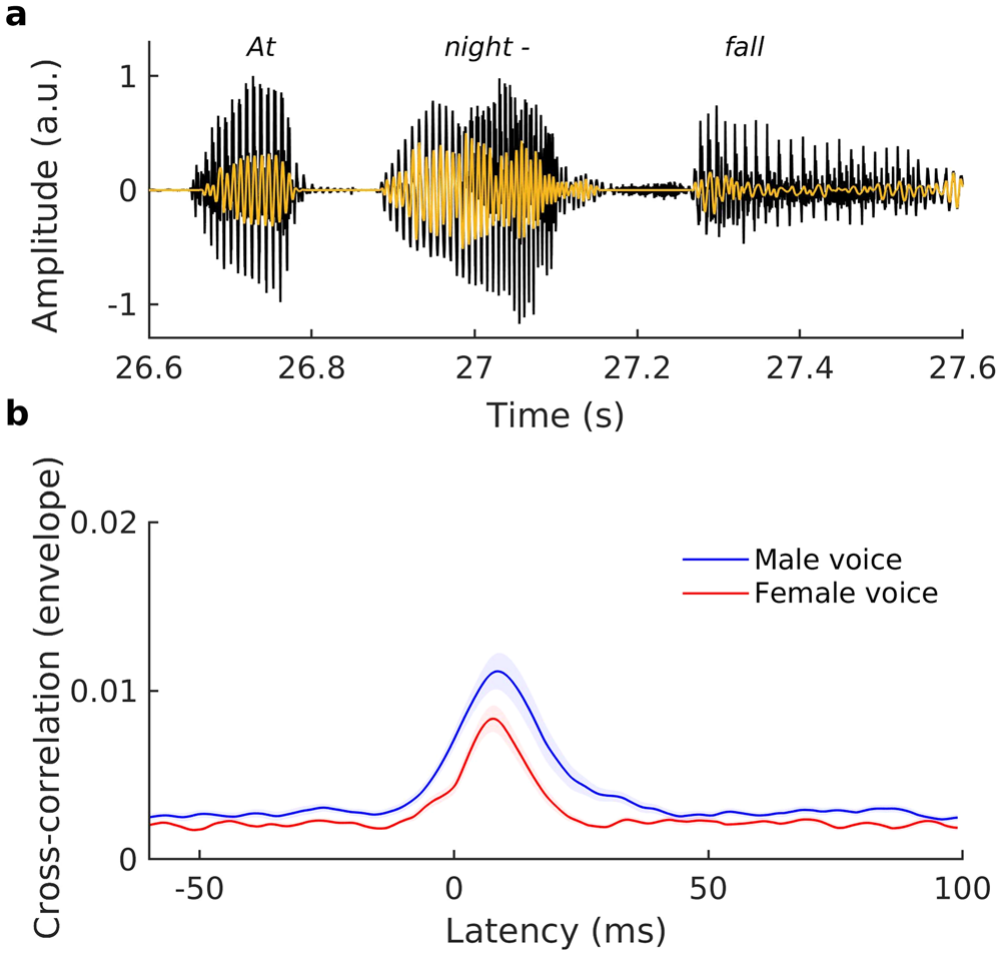
Auditory brainstem response to running speech. (**a**) Speech (black) consists of many voiced parts that are characterized by a fundamental frequency that varies over time. We compute a fundamental waveform (yellow) that, at each time point, oscillates at the fundamental frequency. (**b**) We compute the cross-correlation of the brainstem response with the fundamental waveform to measure the brainstem response to speech when subjects are presented with two competing speakers, a male and a female voice. The envelope of the cross-correlation of the neural recording with the fundamental waveform of the male speaker (blue line: population mean, blue shading: population standard error of the mean) peaks at 8.3 ms. The female voice causes a similar brainstem response, although at a smaller magnitude (red line: population mean, red shading: population standard error of the mean).

To obtain the speech-ABR we computed a fundamental waveform of each voiced part of a speech signal. The fundamental waveform was a temporal signal that, at each time point, oscillated at the fundamental frequency of the voiced speech (Fig. 1a). It was computed using empirical mode decomposition (EMD) of the speech stimuli^30,48^.

We then computed the cross-correlation of the fundamental waveform with the brainstem recording. To this end we band-pass filtered the brainstem response between 100–300 Hz (high-pass filter: FIR, transition band from 90–100 Hz, stopband attenuation −80 dB, passband ripple 1 dB, order 6862; low-pass filter: FIR, transition band 300–360 Hz, stopband attenuation −80 dB, passband ripple 1 dB, order 1054) and compensated for the filter delay. The first ten seconds of each recording were discarded to remove any transient activity. The data were then divided into 40 epochs of three seconds in duration and the remaining data, if any, were discarded. For each segment, the brainstem response was cross-correlated with the corresponding segment of the fundamental waveform as well as with its Hilbert transform, yielding a complex cross-correlation. The complex cross-correlation for an individual subject and a particular condition was obtained by averaging over all corresponding segments. We determined the latency and the amplitude of the peak for each individual.

To determine whether the peak in the cross-correlation obtained for a certain subject and a particular condition was significant, the responses were compared to those of a noise model. For each participant, a null model was computed by cross-correlating the brainstem response to the fundamental waveform of a different story that had not been heard by the participant. The noise floor was determined as the 80^th^ percentile of the complex cross-correlation in the noise model, for latencies from −100 ms to 100 ms. This criterion excluded four and five participants regarding attending to the male voice and attending to the female voice, respectively. Two additional subjects were excluded due to technical problems during the recording (an uncharged battery in one case and loose earphone connection in the other case). One outlier was removed from the measurement of the brainstem response to the female voice when the latter was attended.

To investigate the attentional modulation of the brainstem response to speech, we computed normalized differences. Denote by ${r}_{M}^{(A)}$ the peak amplitude of the complex cross-correlation of the brainstem recording with the fundamental waveform of the male voice, when the male speaker was attended, and by ${r}_{M}^{(I)}$ when the male speaker was ignored. The *relative attentional modulation of the brainstem response to the male voice*, that is, the difference between the brainstem response to the male speaker in the two conditions divided by the average brainstem response, followed as ${A}_{M}=2\frac{{r}_{M}^{(A)}-{r}_{M}^{(I)}}{{r}_{M}^{(A)}+{r}_{M}^{(I)}}$. A positive relative attentional modulation signified a larger brainstem response to the male voice when it was attended, and a negative value implied a larger brainstem response when the male voice was ignored. Analogously, we defined the peak amplitudes ${r}_{F}^{(A)}$ and ${r}_{F}^{(I)}\,$for the cross-correlation of the brainstem recording with the fundamental waveform of the female voice, when it was attended respectively ignored. These coefficients yielded the relative attentional modulation of the brainstem response to the female voice, ${A}_{F}=2\frac{{r}_{F}^{(A)}-{r}_{F}^{(I)}}{{r}_{F}^{(A)}+{r}_{F}^{(I)}}$

### Statistical analysis

The acquired data were checked for normality through the Kolmogorov–Smirnov test. All measurements, that is, the SRTn, noise exposure, ITD threshold, ABR latency shift, MEMR threshold, the brainstem responses to attended speech as well as the relative attentional modulation of the brainstem response to the male and the female voices, followed a normal distribution. We therefore employed the corresponding parametric tests for subsequent hypothesis testing. The results were corrected for multiple comparisons by controlling the false discovery rate (FDR; q) at 10%. We report the FDR-threshold, that is, the largest value *qi*/*m* that satisfies *p*(*i*) ≤ *qi/m*, for the *i*th ordered *p*-value of the *m* performed tests. We employed the FDR correction rather than a family wise error rate such as in the Bonferroni correction due to its greater statistical power^49^.

## Results

**Figure 2:**
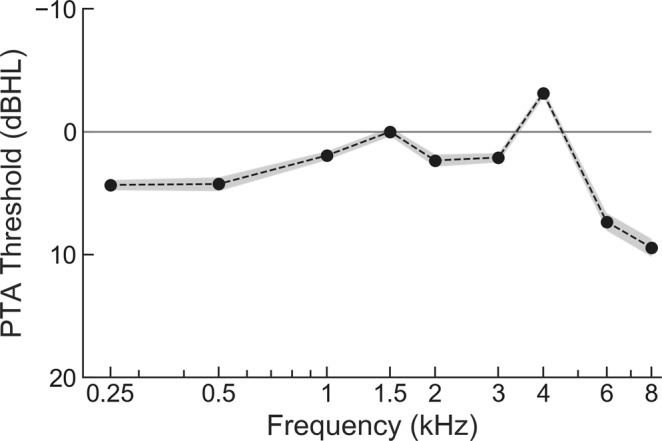
Audiometric thresholds. All participants have normal hearing: their hearing thresholds averaged over the left and right ear are below 10 dBHL (black dots: population mean, grey shading: population standard error of the mean).

The pure-tone audiometry revealed that all participants had pure-tone hearing thresholds better than 20 dB hearing level in both ears at octave frequencies between 250 Hz and 8 kHz (Fig. 2). Sensorineural hearing loss was therefore not present amongst the subjects. However, participants reported a wide range of life-time noise exposure that spanned four orders of magnitude, from 0.005 for the participant with the least exposure to 23 for the participant that reported the highest noise encounter. The geometric mean of the noise exposures across the participants was 6.26. The SRTn varied as well, between −4.2 dB SNR and 0 dB SNR with a population mean of −2.0 dB SNR.

**Figure 3:**
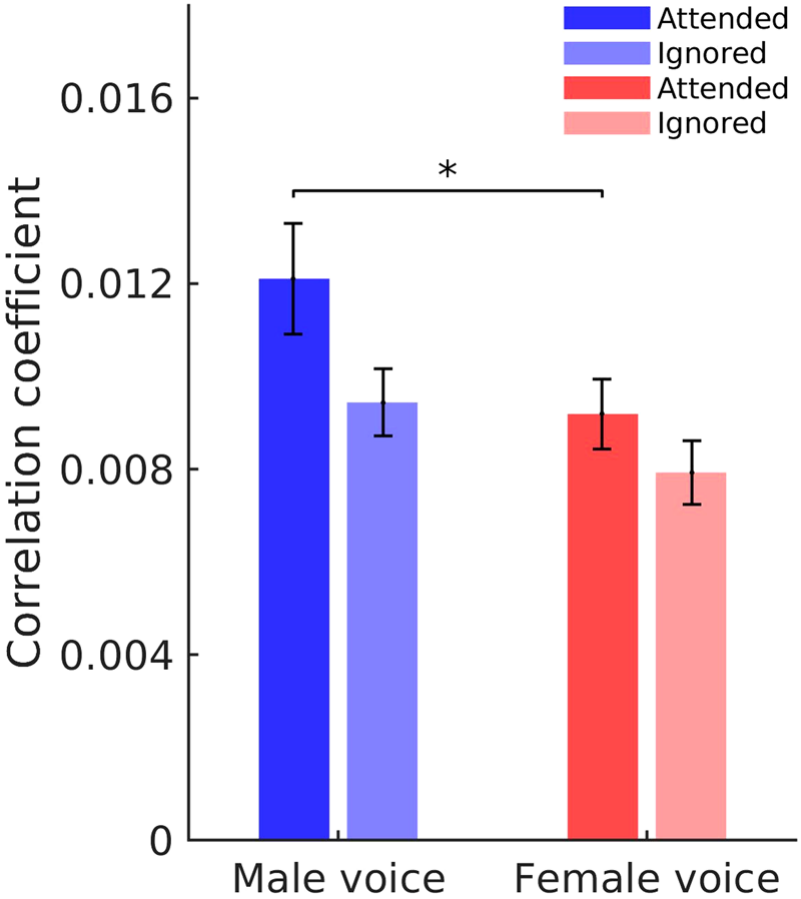
The auditory brainstem response to the male voice is larger when it is attended (dark blue) than when it is ignored (light blue). We observe a similar difference between the brainstem response to the female voice when attended (dark red) and then ignored (light red). Moreover, the brainstem response to the male voice, when attended, is significantly larger than the response to the female voice when attended.

We assessed the auditory brainstem response at the fundamental frequency of running speech by correlating the brainstem recording with the fundamental waveform of the voiced parts of speech (Fig. 1). We obtained peaks in the cross-correlation at an average delay of 8.3 ± 0.3 ms, evidencing the subcortical origin of the response. Furthermore, confirming the results from our previous study, we found that the amplitude at the peak was modulated by selective attention^30,31^. The neural response to the male voice was larger when the subject attended the male speaker, and we found the same attentional effect for the female voice as well (Fig. 3). In particular, the relative attentional modulation of the brainstem response to the male voice, *A*_*M*_, as well as the relative attentional modulation of the brainstem response to the female voice, *A*_*F*_, were significantly greater than zero (population average, *A*_*M*_: *p* = 0.02, *A*_*F*_: *p* = 0.003; two-tailed one-sample Student’s t-tests).

The attentional modulation of the brainstem response was comparable between the male and the female voice. The relative attentional modulation of the brainstem response to the male voice, *A*_*M*_, was indeed not significantly larger than that for the female voice, *A*_*F*_ (*p* = 0.6, two-tailed two-sample Student’s t-test).

Because we sought to investigate a potential correlation between the attentional modulation and the speech-in-noise performance, we were interested in the between-subject *variability* in the attentional modulation. We found that the variability in *A*_*M*_ was significantly larger than the variability in *A*_*F*_ (*p* = 0.0006, one-sided two-sample F-test for equal variances). This difference might have arisen due to differences in the fundamental frequencies between the male and the female voice. We found that the female voice had a mean fundamental frequency of 175 ± 39 Hz, which was significantly different from the male’s fundamental frequency of 123 ± 30 Hz, (mean and standard deviation, *p* = 0.001, two-tailed two-sample Student’s t-test). A higher frequency causes a smaller brainstem response, and we found indeed that the brainstem response to the male voice, when attended, was significantly larger than the brainstem response to the female voice when attended (*p* = 0.05, two-tailed two-sample Student’s t-test)^50–53^.

To investigate which of the different measures of hearing and speech-in-noise listening informed on the subject’s ability to understand speech in noise, we employed multiple linear regression (ordinary least squares) to predict the SRTn from the other variables, that is, from noise exposure, the ITD threshold, the latency shift of wave V of the auditory brainstem response to clicks in noise, the MEMR, and the relative attentional modulation of the brainstem response to the male and the female voice. The data were checked for the conditions that allow linear regression, and all the variables were entered into the model without any selection criteria. In particular, we did not find not find evidence of multicollinearity (all variance inflation factors were below 1.8) or autocorrelation. The model also met homoscedasticity requirement. We corrected for multiple comparisons by controlling the false discovery rate (FDR) at 10%^54^. The only significant factor that remained was the relative attentional modulation of the brainstem response to the male speaker, *A*_*M*_ (Table 1, r^2^ = 0.38, standardised coefficient for *A*_*M*_: 0.461, raw *p*-value for *A*_*M*_: 0.011, FDR-adjusted threshold: 0.017). On the other hand, neither noise exposure nor any of the measures of cochlear synaptopathy were significantly related to speech-in-noise perception.

**Table 1:** We used multiple linear regression to determine which of the auditory measures could explain the variability in speech-in-noise perception.

	Unstandardised coefficients (B)	Standardised coefficients (Beta)	*p* value	95% Confidence Interval for B	
				Lower Bound	Upper Bound
Constant offset	−0.073		0.95	−2.48	2.3
Noise exposure	−0.047	−0.28	0.19	−0.12	0.025
ITD threshold	−0.0053	−0.0018	1.0	−1.30	1.3
ABR latency shift	−55	−0.34	0.10	−121	12
MEMR threshold	0.018	0.20	0.35	−0.020	0.056
Att. mod. male voice (*A*_*M*_)	0.91	0.46	0.011	0.22	1.6
Att. mod. female voice (*A*_*F*_)	−0.028	−0.0082	0.96	−1.2	1.2

After correcting for multiple comparisons (FDR, *q* = 0.1) we obtained only the relative attentional modulation of the brainstem response to the male voice, *A*_*M*_, as a significant predictor; this could explain 38% of the variance in the SRTn.

**Figure 4:**
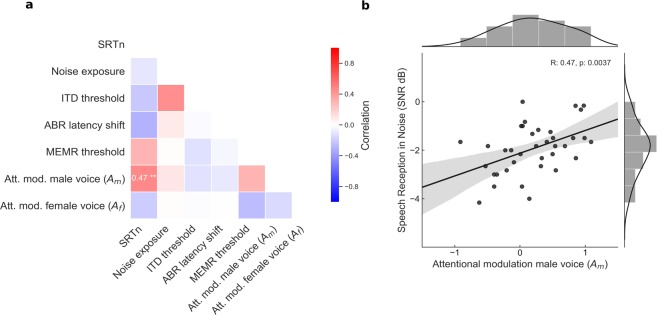
Relation between speech-in-noise perception and the attentional modulation of the brainstem response. (**a**) The only significant pairwise correlation, after correcting for multiple comparisons (FDR; *q* = 0.1), arises between the SRTn and the attentional modulation of the brainstem response to the male voice, *A*_*M*_. There is no significant correlation between the SRTn and noise exposure, or between any of the other auditory measures. (**b**) The attentional modulation *A*_*M*_ can explain 22% of the variation in the speech-in-noise comprehension. The *p-*value (0.0037) remains significant after correcting for multiple comparisons.

We further analyzed the pairwise Pearson correlation coefficients between the different measures that we assessed, that is, between the SRTn, the noise exposure, the ITD threshold, the latency shift of wave V of the auditory brainstem response to clicks in noise, the MEMR, and the attentional modulation of the brainstem response (Fig. 4a). After correcting for multiple comparisons through adjusting for the false discovery rate (FDR) at 10%, we only observed a significant correlation between the SRTn and the attentional modulation of the brainstem response to the male voice (Fig. 4b, *r* = 0.47, raw *p-*value = 0.0037, FDR-adjusted threshold: 0.0048).

## Discussion

Although our volunteers were all young and had normal hearing thresholds, they exhibited variability of life-time noise exposure and of speech-in-noise perception. In particular, the variability in both measures were comparable to those reported in recent studies on young adults^37,55–58^. We showed that a significant portion of the variability in speech-in-noise comprehension could be explained by the individual’s attentional modulation of the brainstem response to speech. In particular, volunteers that exhibited worse speech-in-noise comprehension showed a larger attentional modulation of the brainstem response to the male voice.

Although the correlation between the SRTn and the attentional modulation of the brainstem response to the male speaker was statistically significant even after correcting for multiple comparisons, the raw *p*-value of 0.0037 was only marginally below the corresponding FDR-adjusted significance threshold. The result should therefore be interpreted with caution. However, the large number of potential confounding factors that we have included in our exploratory study has set a high bar for statistical significance, which gives us confidence that the reported relation is indeed significant.

Because the attentional modulation of the brainstem response must involve the corticofugal pathways from the cortex to the brainstem, our finding may indicate that subjects who found it harder to understand speech in noise relied more on this neural feedback mechanism, perhaps to compensate for more central processing deficits. The increased attentional modulation of the brainstem response in subjects who exhibited poorer speech-in-noise comprehension might also have reflected compensation mechanisms at a subcortical level, such as in the inferior colliculus^59–61^.

We note that the larger attentional modulation of the brainstem response in subjects with lower speech-in-noise comprehension parallels previous findings of a negative correlation between the strength of the MOCR and speech-in-noise ability^25,26^. However, the MOCR reflects a broadband reduction of cochlear amplification, whereas the brainstem response that we have studied here is narrowband and may emerge only in the brainstem without involving a change in cochlear amplification. Interestingly, computational work suggests that frequency-specific attentional modulation can improve the comprehension of speech in noise significantly more than broadband modulation^61,62^ which may explain why research on the relation of the MOCR to speech-in-noise comprehension has yielded conflicting results.

The relation between speech-in-noise comprehension and the attentional modulation of the speech-ABR emerged only for the brainstem response to the male, but not the female, voice. The absence of a significant relation between the SRTn and the attentional modulation of the brainstem response to the female speaker may have resulted from a smaller brainstem response to the female than to the male voice. Indeed, we have shown that the smaller brainstem response to the female speaker was linked to less variation in the attentional modulation of the response when compared with the response to the male voice. The smaller brainstem response to the female speaker presumably resulted from the, on average, higher fundamental frequency of the female voice compared to the male one. Higher frequencies, either of a pure tone, in speech tokens or in a musical note, are indeed known to cause a smaller brainstem response, presumably caused by less phase locking in neurons in response to higher frequencies^50–52,63^.

The brainstem response to speech that we measured here emerged at a mean latency of 8.3 ± 0.3 ms. This accords with the latency of the frequency-following response to a pure tone as well as with previously reported latencies of the brainstem response to short speech tokens, and evidences a subcortical origin^50,64^. Although recent MEG and EEG investigations have found that the frequency-following response (FFR) as well as neural responses to the temporal fine structure of speech can also have cortical contributions, we have not observed an additional peak in the brainstem response at a longer latency^65,66^. Such a cortical contribution may nonetheless be present but not measurable in our experiments due to a dominant contribution from the brainstem and due to the relatively broad autocorrelation of the fundamental waveform that limits the temporal resolution and thereby the identification of different components of the neural response^30^. However, the cortical contributions to the FFR are assumed to degrade above 100 Hz and to be absent above 200 Hz^67,68^. The comparable time courses of the brainstem responses to the male speaker, with a fundamental frequency of 123 ± 30 Hz, and of the response to the female speaker, with a significantly higher fundamental frequency of 175 ± 39 Hz, therefore corroborates the absence of a measurable cortical contribution in our recordings.

Although we employed a variety of measures that have recently been proposed for cochlear synaptopathy, no association survived the multiple comparison correction. We therefore found no evidence of this neuropathy amongst our subjects. In particular, we did not find a significant relationship between noise exposure and speech-in-noise perception. Because cochlear synaptopathy has been suggested to lead to a worsened ability to hear in noisy environments, this results suggest the absence of cochlear synaptopathy amongst our volunteers^69,70^. In addition, two objective measures that have recently been suggested to inform on cochlear synaptopathy — the latency change of ABR wave V when listening to clicks in different noise levels as well as the MEMR — did not correlate with noise exposure either. Moreover, we did not find a significant pairwise correlation between any of these behavioural and objective measures. This accords with recent large-cohort studies that have not found a correlation of proposed measures of cochlear synaptopathy to the lifetime noise exposure of the participants or their speech-in-noise perception^37,71,72^. This may suggest that either cochlear synaptopathy has little influence on auditory difficulty or that it has no significant prevalence, at least in normal-hearing people. However, in this study we did not test for high-frequency hearing loss above 8 kHz, which may serve an early indicator of hearing loss at lower frequencies and may indicate cochlear synaptopathy in a broader frequency range^73,74^. Moreover, the measures that we have employed may not have been optimal for detecting cochlear synaptopathy: the latency shift of wave V in noise, for instance, has been recently shown to have only moderate test-retest reliability^55^.

Although we did not find a peripheral origin of the observed variability in speech-in-noise comprehension and in the brainstem measure, to the extent to which our methods capture peripheral processing, future studies are required to tease apart the contributions of bottom-up and top-down impairments to the observed relation between speech-in-noise comprehension and the attentional modulation of the brainstem response to speech. Moreover, our results have been obtained in young adults, and further work is needed to investigate how the attentional modulation of the brainstem response to speech changes with age and how it correlates to speech-in-noise understanding in older listeners.
